# Socioeconomic status moderates the association between patient satisfaction with community health service and self-management behaviors in patients with type 2 diabetes: A cross-sectional survey in China

**DOI:** 10.1097/MD.0000000000015849

**Published:** 2019-05-31

**Authors:** Tao Yin, De-Lu Yin, Feng Xiao, Qian-Qian Xin, Rui-Li Li, Xiao-Guo Zheng, Hui-Min Yang, Li-Hong Wang, Xiao-Yan Ding, Bo-Wen Chen

**Affiliations:** a Department of Health Development, Capital Institute of Pediatrics; b Community Health Association of China, Beijing, China.

**Keywords:** community health service, patient satisfaction, socioeconomic status, type 2 diabetes

## Abstract

The objective of this study was to examine the association between patient satisfaction with community health service (CHS) and self-management behaviors in patients with type 2 diabetes mellitus (T2DM).

In all, 1691 patients with T2DM from 8 community health centers in 5 provinces in China participated in the present study. The dependent variables included 4 measures of self-management behaviors: regular self-monitoring of blood glucose (SMBG), prescribed medication adherence, recommended dietary changes, and regular exercise. The independent variable was patient satisfaction with CHS. Multivariable logistic regression models were performed to examine the association between patient satisfaction with CHS and self-management behaviors.

The mean satisfaction score in the participants was 3.14 (out of a maximum of 5). After adjusting for covariates including demographic factors, health status, health knowledge, and socioeconomic status (SES), diabetic patients with high CHS satisfaction had better medication adherence (odds ratio [OR] 1.25, 95% confidence interval [CI] 1.02–1.55), increased exercise management (OR 1.19, 95% CI 1.06–1.35), and more SMBG (OR 1.16, 95% CI 1.03–1.32); all these associations varied across SES groups. The association between satisfaction and medication adherence was significant among participants younger than 65 years with lower education (OR 2.15, 95% CI 1.37–3.37), income (OR 1.62, 95% CI 1.13–2.32), and lower-status occupations (OR 1.69, 95% CI 1.16–2.47). Among participants younger than 65 years and had lower education attainment, the association between satisfaction and diet management was observed. There were positive associations between satisfaction and regular exercise among subgroups of participants younger than 65 years, except for lower education group. A significant association between satisfaction and SMBG among participants ≥65 years old, who also had lower SES and higher-status occupations, was also observed.

The study findings suggested that T2DM patient satisfaction with CHS was moderate. High satisfaction with CHS indicated better medication adherence, exercise management, and SMBG, and these associations varied by SES.

## 1 Introduction

Diabetes is a rapidly increasing epidemic and has been a primary public health issue. In 2015, 415 million people aged 20 to 79 years were estimated to have diabetes, and this number is expected to rise to approximately 642 million by 2040.^[1]^ Type 2 diabetes mellitus (T2DM) accounts for about 85% to 95% of all diabetes cases in developed countries and is also a serious public health problem in China. A national survey from mainland China reported that the estimated prevalence of total diagnosed and undiagnosed diabetes is 10.9%.^[2]^

Over the past decade, integrated strategies have been used for diabetes treatment, in which health care, such as patient education and self-management, has been emphasized.^[3]^ On the individual level, ongoing self-management behavior (SMB) is significant for T2DM patients’ health. Self-management behaviors have multiple components, such as self-monitoring blood glucose (SMBG), medication adherence, diet management, and regular physical exercise.^[3]^ A meta-analysis focused on the effects of SMB (in both group and individual level) reported that SMB can improve glycemic control.^[4,5]^

Although treatment for diabetes has been much improved, there was still gap between optimal evidence-based medicine and actual practice.^[6]^ Patient self-management plays key role in improving the quality of life.^[7,8]^ It is complex and has many influencing factors such as socioeconomic status (SES), culture, health, and social policies.^[6]^ Lower SES was associated with poor adherence to prescribed medication and SMBG.^[9,10]^ The importance of patient satisfaction with primary care is increasingly being recognized due to its effect on prescribed medication adherence, and also on life quality promotion.^[11–15]^ Previous studies supported the idea that patients who were satisfied with their health care provider had better adherence and glycemic control, and patients with high satisfaction toward community healthcare provider were also observed receiving better ongoing care and having more effective SMB.^[8,13]^

In urban China, physicians who work in community health centers play the role of primary care provider. However, the association between patient satisfaction toward community health service (CHS) and diabetes SEM is still unclear in mainland China. In addition, whether SES modifies this relationship has not been well studied either. The objective of the present study is to investigate the association between patient satisfaction with CHS and SMB in patients with T2DM, and also the modification effect of SES.

## 2 Methods

### 2.1 Study design and population

Face-to-face structured questionnaire interviews were conducted in this survey. All interviewers completed a training program, in which detailed instructions for questionnaire administration were given to them. A multistage stratified cluster sampling method was used to select participants. In the first stage, 5 provinces were selected from the mainland China based on geographic distribution; in the second stage, 1 or 2 community health centers were selected from each province based on the population size of each province (1 center in Anhui, 1 in Tianjin, 2 in Shandong, 2 in Sichuan, and 2 in Guangdong); in the third stage, residents in the selected community who met the study criteria were all invited to participate.

All participants were carefully informed that they could refuse to answer any question before the formal survey. The inclusion criteria for participants were: no less than 18 years old; being diagnosed with T2DM in accordance with the guidelines of World Health Organization^[16]^; resident in the study area for no less than 2 years; and be able to provide written informed consent. Patients were excluded that were pregnant, had psychological problems or physical disabilities, or who were unable to complete the questionnaire.

Questionnaire questions were established based on literature review^[17]^ and expertise consultation. After the pilot test, the final version of the questionnaire was developed comprised of information on sociodemographic characteristics, basic physical information, knowledge about T2DM, and information on self-management behaviors. The response rate was 92% and the main reason for nonparticipation was that the patient had migrated to the community less than 2 years earlier.

### 2.2 Measures

The dependent variables included 4 binary measures of the following SMB: regular self-monitoring of blood glucose (SMBG), taking medications following physician's recommendations (defined as taking medications exactly as the physician's prescription, dose unchanged, and no more than twice per month in the situation of forgetting to take the medication), making the recommended dietary changes (defined as less sugar, less fat and a high-fiber diet), and taking regular exercise (regular exercise was defined as at least 20 minutes moderate physical activity each time and at least three times per week). The definition of regular SMBG was based on patients’ answers to the question: “How many times did you test your blood glucose per week during the past half year?” Because only 75 (4.4%) responders reported SMBG more than once a day, regular SMBG was defined as testing blood glucose at least once a week. The question, “Have you taken the following actions to control your blood glucose during the last half year? (eg, food consumption control, adherent to medication therapy, body weight control, or physical exercises),” was used to assess medication adherence, diet management, and regular exercise. The time span of 6 months guarantees the relatively fixed behavior styles and less recall bias.

The independent variable was patient satisfaction with CHS, which was measured with a 5-point Likert scale (a score of 1 represents the lowest degree of satisfaction and a score of 5 represents the highest degree of satisfaction), based on the question: “How satisfied are you with community health service during the past half year?” The control variables were age (<65 years/≥65 years), sex (male/female), being overweight or obese (overweight: 24 kg/m^2^ ≤ body mass index [BMI] ≤28 kg/m^2^; obesity: BMI ≥28 kg/m^2^),^[18]^ duration of diabetes from being diagnosed (more than 5 years/less than 5 years), SES, and diabetes knowledge. SES was measured by levels of education, income, occupation and health insurance type. According to each SES variable, patients were divided into either lower or higher group based on China's context. Education level was divided into 2 categories: no more than primary school or at least junior school. The lower income group included participants with a per-capital annual income below 18 thousand RMB (1 RMB = 0.15 USD). For the occupation variable, participants were divided into retired, farmer, worker, or businessperson groups. The higher insurance group included patients covered by urban employee basic medical insurance (UEBMI), state-free medical care, or labor insurance, which all have higher compensation levels. Low insurance group included patients covered by urban resident medical insurance, new rural cooperative medical scheme (NRCMS), other health insurance, or uninsured. Diabetes knowledge was assessed by whether the subject was able to answer the following questions correctly: “What is normal fasting plasma glucose (FPG) level?”; “What are the therapeutic principles of T2DM?” “What are the potential side effects of antidiabetic drugs?”; and “How is hypoglycemia should be treated?”

### 2.3 Statistical approach

Baseline characteristics were evaluated after controlling for age group, because previous studies demonstrated an age difference in satisfaction and self-management behavior.^[19,20]^ Preliminary analyses on age differences were carried out using *t* test on continuous variables and chi-square test on categorical variables.

Multivariable logistic regression models were used to examine the association between patient satisfaction with CHS and SMB. An unadjusted model was fitted and then gradually other models adjusted for demographic characteristics (age, sex), health status (body weight, duration of diabetes), diabetes knowledge, and SES (educational attainment, household income per capita, and occupation) to estimate the association between satisfaction and SMB.

The modification effect of SES on the association between satisfaction and SMB was also assessed. A series of models were fitted to examine this mediating effect. A *P* value less than .05 was considered statistically significant. Stata version 14.0 for Windows (Stata Corp, College Station, TX) was used for statistical analyses.

### 2.4 Ethics approval and consent to participate

Ethical approval is not required for conducting this type of health services survey in China (reference file # Science and Education Department of Ministry of Health [2007] 17# http://www.moh.gov.cn/mohbgt/pw10702/200804/18816.shtml). However, informed consent of respondents was obtained. A detailed explanatory statement was given to respondents describing the study, which highlighted that their participation was voluntary and no identifiable personal data would be collected.

## 3 Results

### 3.1 Respondent characteristics

Participant characteristics are present in Table 1. The final sample consisted of 1691 individuals after excluding patients with missing data. Among them, 34.48% of the participants were men; 54.29% of all the participants got high-level education; and 35.25% had an annual household income above 18 thousand RMB. More than half (55.23%) of the subjects were retired, and 53.58% had UEBMI, state-free medical care, or labor insurance; 46.30% of the participants were overweight or obese, and 54.29% reported having been diagnosed as diabetes for more than 5 years. For participants’ disease-related knowledge levels, 71.67% knew what constitutes a normal FPG level; 78.95% knew the therapeutic principles of T2DM; 56.36% knew the side effects of antidiabetic drugs; and 69.19% knew how to treat hypoglycemia.

**Table 1:**
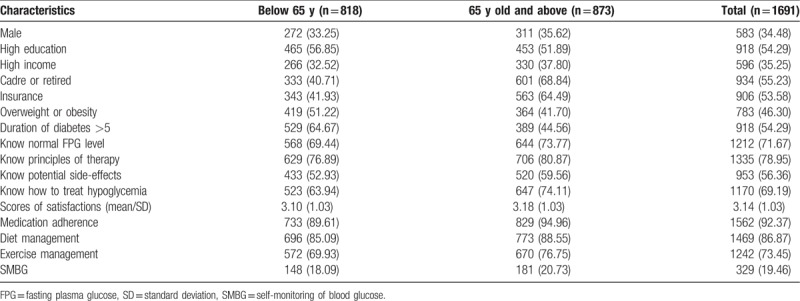
Characteristics of participants (n/%).

The average score of satisfaction was 3.14, and there was no significant difference among age groups (*P* = .134). Among the participants, 19.46% reported that they tested their blood glucose more than once a week, and there was no statistical significance among age groups on SMBG (*P* = .171). The reported percentages for medication adherence, diet management, and exercise management were 92.37%, 86.87%, and 73.45%, respectively. In contrast with SMBG, among the older-age group, the proportions of medication adherence, diet management, and exercise management were significantly higher (all *P* < .05).

### 3.2 Association between satisfaction and self-management behaviors

Table 2 presents a series of binary logistic regression outputs for the associations between satisfaction and SMB. An unadjusted model was fitted first and then gradually adjusted for other covariates. After adjusting for demographic, health status, health knowledge, and SES, diabetic patients with high CHS satisfaction were more likely to be adherent to their medication (odds ratio [OR] 1.25, 95% confidence interval [CI] 1.02–1.55), exercise management (OR 1.19, 95% CI 1.06–1.35), and SMBG (OR 1.16, 95% CI 1.03–1.32). For exercise management and SMBG, the estimated values decreased slightly after the adjustments of covariates: OR changed from 1.29 to 1.19 and from 1.23 to 1.16, respectively. Before SES was considered as a covariate, there was no significant association between patient satisfaction and medication adherence. No significant association between satisfaction and adherence to diet management was observed.

**Table 2:**
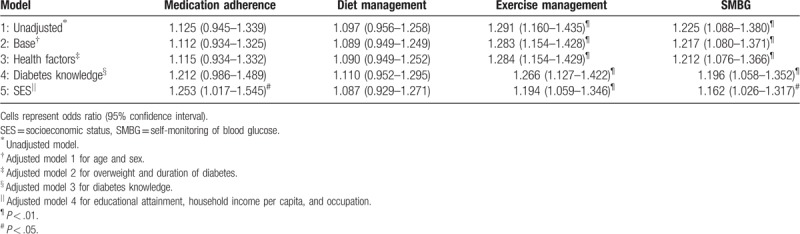
The association of satisfaction and self-management behavior.

### 3.3 SES as a modifier of the association between satisfaction and self-management behaviors

Table 3 shows the effect of SES in modifying the association between satisfaction and self-management behaviors. The stratification analysis indicated positive association between satisfaction and medication adherence among participants <65 years old who had lower status on education (OR 2.15, 95% CI 1.37–3.37), income (OR 1.62, 95% CI 1.13–2.32), and occupation type (OR 1.69, 95% CI 1.16–2.47). Similarly, for diet management, the association only existed in participants <65 years old with lower education attainment (OR 1.53, 95% CI 1.12–2.10). There were positive associations between satisfaction and regular exercise among all the low SES groups <65 years old, except the lower-education group. Unlike the other SMB being measured, a significant association between satisfaction and SMBG was found among participants ≥65 years old with lower status on education, income, and insurance, but higher status of occupations (OR 1.53, 95% CI 1.16–2.02; OR 1.65, 95% CI 1.29–2.11; OR 1.82, 95% CI 1.19–2.78; OR 1.33, 95% CI 1.08–1.64; and OR 1.22, 95% CI 1.00–1.49, respectively).

**Table 3:**
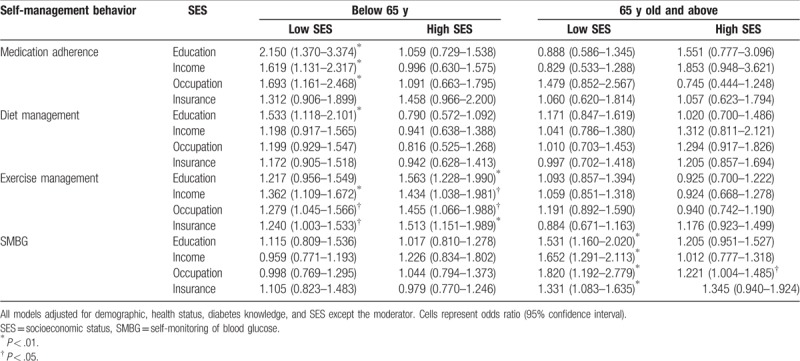
The association of satisfaction and self-management behavior in different SES groups.

## 4 Discussion

An average satisfaction score of 3.14 was estimated (out of a maximum possible score of 5). Participants who had high satisfaction with CHS mostly had a positive association with medication adherence, exercise management, and SMBG, and all these associations varied across the specific SES and age groups.

Only 1 short question was used to measure the overall satisfaction with CHS, and the results indicated that T2DM patients were not fully satisfied. In a study from Jilin Province in China, 81.3% of people reported feeling satisfied with their primary care service.^[21]^ Another study from Hubei province in China found an overall moderate level of patient satisfaction with CHS (mean score = 3.0, out of a maximum possible score of 5).^[22]^ Because the definitions of patient satisfaction used for these 2 studies were not identical, comparisons should not be made due to the differences in the studies. The study sample covered a wide range of Chinese provinces, and was composed of provinces in the eastern, central, and western regions of China. A relatively moderate level of overall CHS satisfaction (3.14 out of 5) was found across the study settings.

The patients in the study exhibited good performance on SMB, with the exception of SMBG, which was consistent with another recent study.^[7]^ Blood glucose self-monitoring equipment and supplies are available in China, yet diabetic patients, especially those with lower SES, are unable to afford the equipment and testing strips, which are not covered by health insurance.^[23]^ Furthermore, patients who do own equipment perform self-testing less frequently as recommended.^[24]^

After adjusting for demographic characteristics, health status, diabetes knowledge, and SES, the study found that diabetic patients with high CHS satisfaction were more likely to adherent to their medication, exercise management, and SMBG protocols. These associations are consistent with those seen in other studies.^[25–27]^ Patient satisfaction is an important aspect of quality health care. A previous study indicated that the quantity and quality of healthcare service was positively associated with patient satisfaction.^[28]^ This satisfaction was correlated with self-efficacy: patients who had strong self-efficacy were more likely to take responsibility for their health by adopting self-management behavior and were also more likely to be satisfied with their health service. Additionally, good provider–patient communication can improve patient knowledge, confidence, and attitude about diabetes; all of which can positively influence health behaviors.^[29,30]^

The study also observed that the relationship between satisfaction and behavior was only present for specific SES and age groups. These findings may broaden the understanding of the association between patient CHS satisfaction and self-management behavior. The moderated relationship for medication adherence, exercise management, and diet was only present among patients <65 years old who also had lower SES, lower education attainment, and all SES, respectively. Some studies have shown that elderly patients and those with higher SES status exhibited better self-management behavior.^[10,20,31]^ Previous study supported that people with lower SES status reported less confidence in the ability of self-management of diabetes,^[25]^ and this may be attributable to ineffective blood glucose control. People with higher SES had higher levels of access to health services comparing with those with lower SES. For SMBG, the association was only present among patients above 65 years old who had lower SES, possibly because it is more difficult for older people with lower SES than younger people with higher SES, to pay the fees of toolkits or equipment for blood glucose monitoring.

The questionnaire was developed based on literature review and expertise consultation. Members of the expert panel were drawn from CHS administration centers, the Community 283 Health Association of China, universities, and local CHS centers. All members had more than 10-year work experience in CHS or relevant areas with (over 80%) expertise in population survey and questionnaire design. This enabled the content of the questionnaire to cover and measure the main problems concerning SMB of T2DM patients in CHS centers in the study sites. Additionally, to make the questionnaire feasible and understandable to interviewers, and also interviewees, pilot interviews were conducted and a revised version based on the feedback was established. Therefore, the scientific and practical factors were both considered in the establishment of questionnaire.

The limitations of the present study should also be acknowledged: the information on self-management behavior was based on self-reported data, which may have been subject to recall and self-report biases; because the study design is cross-sectional, it is hard to identify causal effect; it is difficult to identify which aspect of satisfaction is most important for self-management behavior because it was measured only by 1 question; and because our questionnaire questions were established based on literature review and expertise consultation but not a validated tool, the validation of this questionnaire should be further considered and tested in the future, and a more feasible questionnaire should be designed based on the existing instruments measuring self-management of diabetes such as the Diabetes Management Self-Efficacy Scale (DMSES) or Diabetes Self-management Assessment Report Tool (D-SMART).^[32,33]^ Despite these limitations, the study was the first one to assess the association between patient satisfaction with CHS and self-management behavior among T2DM patients in China, and the first one to evaluate the moderating role of SES as well.

## 5 Conclusions

This study suggested that T2DM patient satisfaction of community health was moderate, and high CHS satisfaction was positively associated with medication adherence, and the adherence of exercise management and SMBG. These associations varied according to specific SES groups. Strategies to improve patient satisfaction should be tailored to focus on target populations with poorer abilities or efficacy to adopt health behaviors.

## Acknowledgments

The authors appreciate all the participants, researchers, and healthcare workers in the 8 community health centers for their support in this study. We also thank Huijing He for her efforts on the manuscript revision and Yvonne Li for language editting.

## Author contributions

T.Y. designed the study, participated in the field work, performed the statistical analysis, and wrote the manuscript. D.-L.Y. and F.X. contributed to conduct the study, collect research data, and figures and paper preparation. Q.-Q.X., R.-L.L., X.Z., H.-M.Y., L.-H.W., and X.-Y.D. participated in the interpretation of data and revised the paper. B.C. conceived and designed the survey. All authors read and approved the final manuscript.

**Conceptualization:** Feng Xiao, Bo-wen Chen.

**Data curation:** Qian-qian Xin, Rui-li Li, Hui-min Yang.

**Formal analysis:** Xiao-yan Ding.

**Funding acquisition:** Bo-wen Chen.

**Investigation:** Qian-qian Xin, Rui-li Li, Xiao-guo Zheng, Hui-min Yang, Li-hong Wang.

**Methodology:** Qian-qian Xin.

**Resources:** Bo-wen Chen.

**Software:** Xiao-guo Zheng, Hui-min Yang, Xiao-yan Ding.

**Supervision:** De-lu Yin, Feng Xiao, Bo-wen Chen.

**Writing – original draft:** Tao Yin.

**Writing – review & editing:** Tao Yin.
